# Snail1 is required for the maintenance of the pancreatic acinar phenotype

**DOI:** 10.18632/oncotarget.6785

**Published:** 2015-12-29

**Authors:** Jordina Loubat-Casanovas, Raúl Peña, Núria Gonzàlez, Lorena Alba-Castellón, Santi Rosell, Clara Francí, Pilar Navarro, Antonio García de Herreros

**Affiliations:** ^1^ Programa de Recerca en Càncer, Institut Hospital del Mar d'Investigacions Mèdiques (IMIM), 08003 Barcelona, Spain; ^2^ Servei d'Oncologia Mèdica, Hospital del Mar, 08003 Barcelona, Spain; ^3^ Escola Superior Infermeria del Mar, Universitat Pompeu Fabra, 08003 Barcelona, Spain; ^4^ Departament de Ciències Experimentals i de la Salut, Universitat Pompeu Fabra, 08003 Barcelona, Spain

**Keywords:** Snail1, pancreatic mesenchymal cells, fibroblast activation, pancreas physiology, acinar-ductal metaplasia

## Abstract

The Snail1 transcriptional factor is required for correct embryonic development, yet its expression in adult animals is very limited and its functional roles are not evident. We have now conditionally inactivated Snail1 in adult mice and analyzed the phenotype of these animals. Snail1 ablation rapidly altered pancreas structure: one month after Snail1 depletion, acinar cells were markedly depleted, and pancreas accumulated adipose tissue. Snail1 expression was not detected in the epithelium but was in pancreatic mesenchymal cells (PMCs). Snail1 ablation in cultured PMCs downregulated the expression of several β-catenin/Tcf-4 target genes, modified the secretome of these cells and decreased their ability to maintain acinar markers in cultured pancreas cells. Finally, Snail1 deficiency modified the phenotype of pancreatic tumors generated in transgenic mice expressing c-myc under the control of the elastase promoter. Specifically, Snail1 depletion did not significantly alter the size of the tumors but accelerated acinar-ductal metaplasia. These results demonstrate that Snail1 is expressed in PMCs and plays a pivotal role in maintaining acinar cells within the pancreas in normal and pathological conditions.

## INTRODUCTION

Snail1 is a transcriptional repressor that triggers the epithelial-to-mesenchymal transition (EMT), which enables epithelial cells to acquire migratory properties [1]. It also provides epithelial cells with higher resistance to apoptosis and induces some properties of cancer stem cells [1, 2]. Molecularly, Snail1 acts by repressing the expression of target genes, such as E-cadherin [3, 4], and by inducing mesenchymal genes by releasing the inhibition of transcriptional activators by E-cadherin [5]. For instance, Snail1 enhances β-catenin translocation to the nucleus and the expression of β-catenin/Tcf-4 target genes [6]. Besides releasing E-cadherin inhibition, Snail1 plays an active role by directly interacting with β-catenin [7], thereby potentiating its transcriptional activity.

In cultured fibroblasts, Snail1 expression is not constitutive and is dependent on serum [8, 9]. Several studies have also shown that Snail1 is rapidly induced by cytokines, such as TGF-β, both in epithelial and mesenchymal cells [10–12]. Genetic depletion in murine embryonic fibroblasts or in fibroblast cell lines has revealed that it is required for expressing genes essential for invasion, such as membrane type-1 matrix metalloprotease [9], and for the induction by TGF-β of markers of activated fibroblasts, such as β1 integrin [12]. Indeed, Snail1-depleted fibroblasts show an incomplete response to this cytokine, suggesting that fibroblasts cannot be fully activated in the absence of Snail1 [12].

Snail1 is widely expressed during embryonic development; Snail1-defective embryos are not viable, since gastrulation is not carried out properly [13]. In contrast, Snail1 expression is very limited in adult animals in non-pathological conditions [8, 14]. However, it is expressed in several types of tumors with an epithelial or a mesenchymal origin [15, 16]. Although these studies have been limited by the poor reliability of Snail1 antibodies, expression of Snail1 appears to be normally restricted to fibroblast cells in the stroma, located in areas of invasion [8, 14]. Accordingly, Snail1 expression has been detected in carcinoma-associated fibroblasts [17].

Due to the expression of Snail1 in neoplasm and its demonstrated role *in vitro* in triggering EMT, cellular invasion and chemoresistance, it has been proposed as a putative target for therapeutic intervention (for instance, see [18]). To analyze the side-effects of this inhibition as well as the role of Snail1 in adult mice, we used a transgenic animal in which Snail1 expression is eliminated upon tamoxifen injection. Our results indicate that Snail1 is expressed in pancreatic mesenchymal cells and plays an unsuspected role in pancreas homeostasis.

## RESULTS

### Snail1 depletion affects pancreas morphology

**Figure 1:**
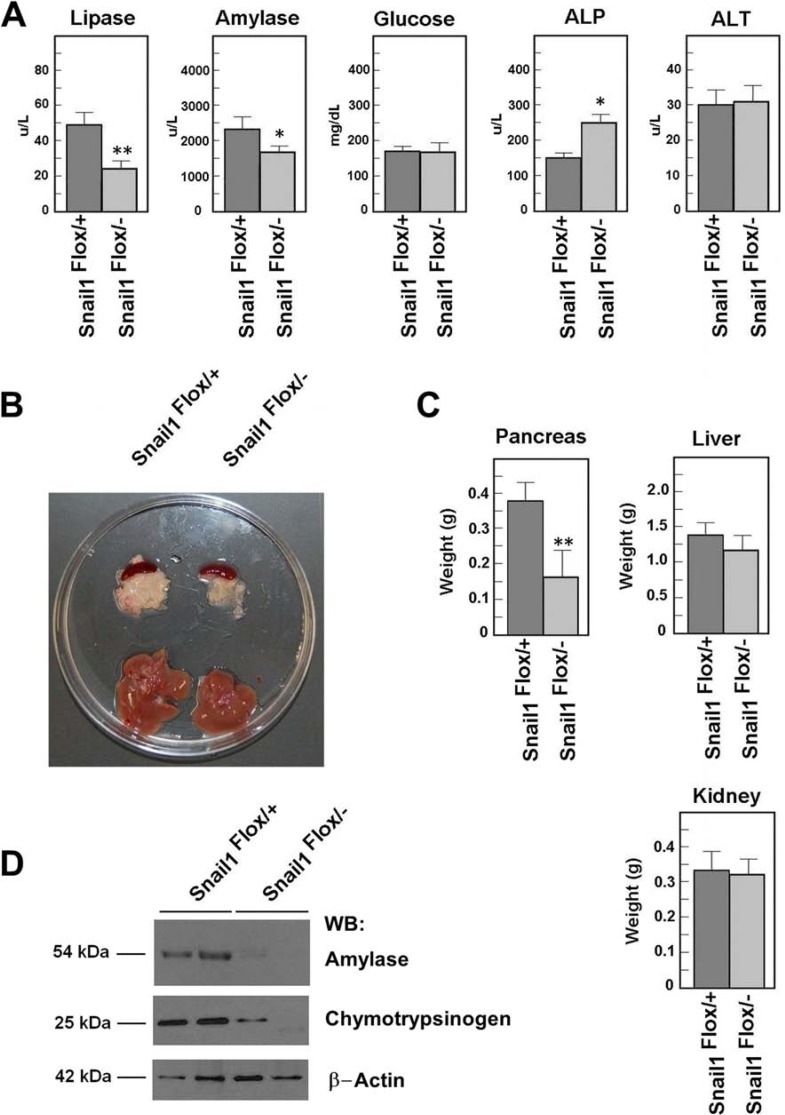
Snail1-depleted mice present a smaller pancreas Snail1^Flox/+^ or Snail1^Flox/–^ mice were treated with tamoxifen (TAM) as indicated in the Materials and Methods. Animals were analyzed four weeks later. (**A**). The determinations of lipase, amylase, alkaline phosphatase (ALP) and transaminase (ALT), or glucose were performed in serum as indicated. (**B**). A representative photograph of the pancreas and liver of control or Snail-deficient animals is shown, as well as the weight average (± SD) of the pancreas, liver, and kidney (**C**). Two samples corresponding to homogenates of control or mutant pancreas were prepared and analyzed by Western blot with the indicated antibodies (**D**). One asterisks indicate that the differences are significant with a *p* < 0.05; two asterisks, with a *p* < 0.01.

We generated a mouse with Snail1 null and Snail1 floxed alleles combined with tamoxifen-inducible Cre recombinase, under the control of the ubiquitously active β-Actin promoter (β-Act/Cre-ER; [12]). Snail1 was depleted by injecting tamoxifen (TAM) in Snail1^Flox^/^−^, β-Act-Cre-ER mice or Snail1^Flox^/^+^, β-Act-Cre-ER control littermates. We analyzed the relevance of Snail1 expression in two-month-old animals. Two weeks after TAM injection, murine stools from Snail1^Flox^/^−^ showed the presence of fat, suggesting altered lipase function in the gastrointestinal tract. At four weeks after TAM injection, a serum analysis demonstrated that lipase activity was indeed downregulated in Snail1-depleted animals (Figure 1A). The serum levels for albumin, alanine aminotransferase, urea, phosphate, Ca^2+^, Na^+^, and K^+^ were similar between the two populations; only amylase was significantly decreased, whereas alkaline phosphatase was upregulated (Figure 1A). However, the levels of aminotransferases were similar in control and Snail1-depleted mice, indicating that the hepatic function was not altered. Glucose levels were not significantly different (Figure 1A).

At four weeks after TAM injection, Snail1-depleted animals had a pancreas with a smaller size (Figure 1B) and lesser weight (Figure 1C) than the control animals. Further, expression of exocrine function markers, such as amylase or chymotrypsinogen, was also considerably reduced in the pancreas from Snail1-depleted animals (Figure 1D). No decrease in size was observed in other organs; for instance, four weeks after Snail1 depletion, liver weight was only slightly and not significantly lower (Figure 1C), while colon, small intestine, and kidneys were normal. A histological analysis of these organs did not reveal abnormalities (Supplementary Figure S1).

**Figure 2:**
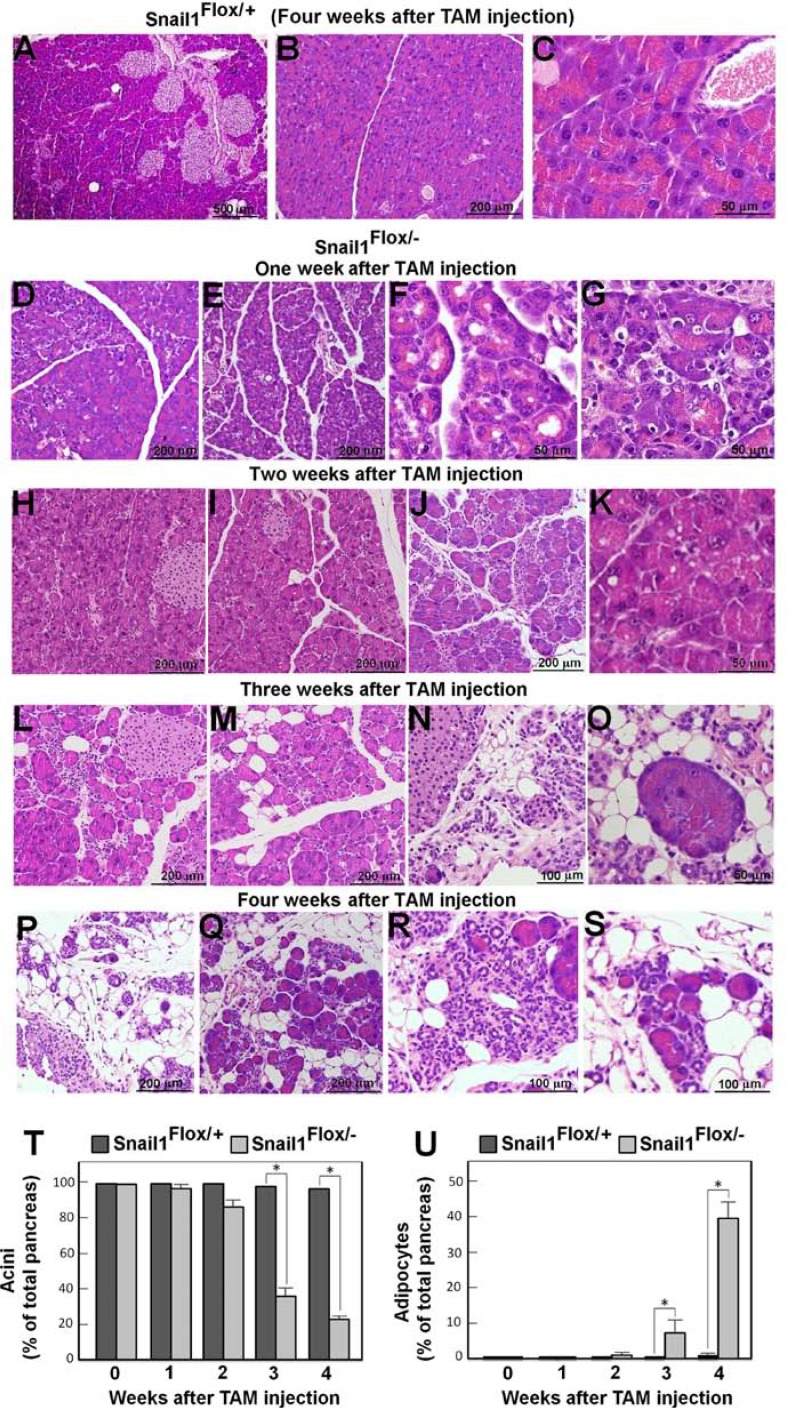
Snail1 depletion promotes the rapid replacement of acini by adipose tissue The figure shows sections of control (Snail1^Flox/+^) (panels **A–C**) or Snail1-deficient (Snail1^Flox/–^) (**D–S**) mice stained with hematoxylin-eosin at different times after TAM injection. The magnification is indicated. The morphology of the pancreata of Snail1^Flox/–^ mice prior to TAM treatment is similar to those shown in panels A–C. Samples were taken one (D–G), two (H–K), three (L–O) or four (A–C, P–S) weeks after TAM administration. In panels (**T** and **U**) the relative area of acinar cells or adipocytes with respect to the total pancreas area was determined in several representative sections. The average (± SD) of five different experiments is presented at different times after TAM injection. When not shown, SD was lower than 1%. The asterisk indicates that the differences are significant with *p* < 0.05.

In contrast to the control mice that exhibited a normal phenotype in the pancreas after TAM administration (Figure 2A–2C), Snail1-depleted animals showed histological alterations in this organ. Although without gross alterations, as early as one week after TAM injection the pancreas looked less compact, with a greater separation among the acini, which had also partially lost their structure (Figure 2D–2G). Apoptotic cells, visualized by analyzing active caspase-3 expression, were observed and mainly corresponded to the acinar cells (Supplementary Figure S2). Apoptosis was not detected in control pancreas (Supplementary Figure S2A). Further, in the Snail1-depleted pancreas, apoptotic cells were present mainly at one week post-TAM (Supplementary Figure S2B–S2G), decreasing at later time points (Supplementary Figure S2H–S2I). An immunofluorescence analysis confirmed that apoptotic cells were acinar cells, since they co-express amylase and not CK19, a specific marker of ductal cells (Supplementary Figure S3).

Two weeks after Snail1 depletion (Figure 2H–2K), most part of the pancreas looked still relatively normal although some areas displayed a detectable loss of acini and an enrichment in ductal cells (Figure 2J), also determined by analyzing CK19 (Supplementary Figure S4, panels G–L). Small vesicles were often detected inside the acinar cells (Figure 2K). Three weeks after TAM injection the pancreas was totally disorganized (Figure 2L–2O) and the loss of the acini was more evident (Figure 2N); ductal cells constituted the majority of the remaining epithelial cells (Supplementary Figure S4K–S4L). Presence of adipocytes became evident at three weeks (Figure 2L–2O) and they accumulated at four weeks (Figure 2P–2S). A quantitative analysis indicated that four weeks after Snail1 depletion approximately 40% of the total pancreas area was occupied by adipocytes (Figure 2U). Actually at this time pancreata presented a histological phenotype resembling pancreatitis. Some acini survived in all the animals even at four weeks after TAM injection (Figure 2O, 2Q, and 2S), corresponding to approximately the 20% of the pancreas; these acini were shown to potently express amylase (Supplementary Figure. S4, panels A–F). Islets remained unaffected, in agreement with the normal glucemia in these animals. Islet architecture was normal, and endocrine cells from Snail1-depleted animals showed a similar expression as insulin and glucagon as from control pancreas (Supplementary Figure S5).

These results indicate that Snail1 depletion in adult mice causes acini to be lost and eventually replaced by adipocytes in the pancreas.

### Snail1 is expressed in pancreatic mesenchymal cells

To determine if the effect of Snail1 depletion in acinar homeostasis was cell-autonomous, we generated another murine line using the PTF1/p48 promoter to drive Cre-ER expression. The PTF1/p48 transcriptional factor is specifically expressed in pancreatic acinar cells [19]. Four weeks after TAM injection, PTF1/p48–Cre-ER, Snail1 ^Flox/–^ animals showed no abnormalities in the pancreas and were comparable to the control Snail1^Flox/+^ animals (Supplementary Figure S6). This result indicates that loss of acinar cells depends on a lack of Snail1 expression in a different cell type.

**Figure 3:**
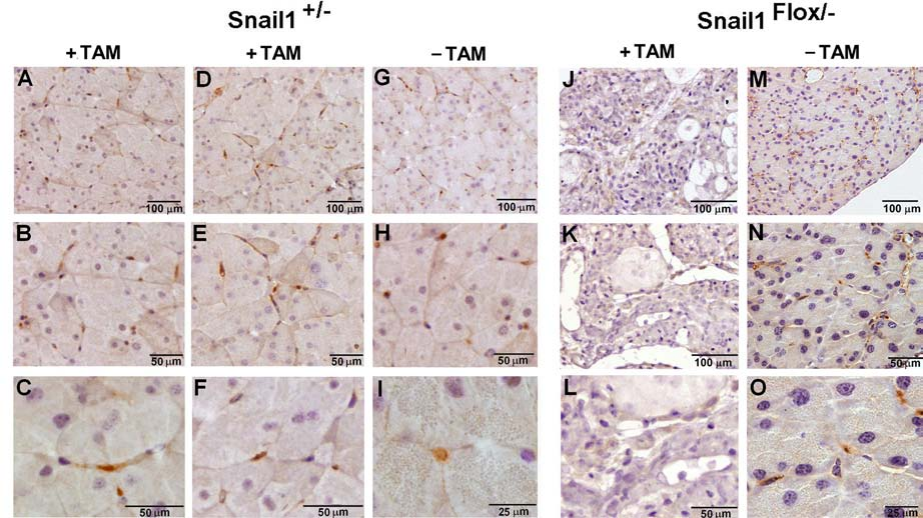
Snail1 is expressed in mesenchymal cells in the pancreas Expression of Snail1was assessed in the pancreas of Snail1^Flox/+^ (**A–I**), or Snail1^Flox/–^ (**J–O**) mice treated with tamoxifen when indicated for one week. The bars indicate magnification.

We then determined the presence of Snail1 in the pancreas by immunohistochemistry, using murine embryos or human placenta as positive controls (Supplementary Figure S7). As seen in Figure 3, Snail1 was not detected in acinar or ductal cells but rather in cells with an elongated phenotype that embrace the acini; we called these cells pancreatic mesenchymal cells (PMCs). Snail1-positive cells were also observed in the vicinity of the islets. Snail1 was localized in the nucleus, as expected for a functional transcription factor, and was also detected in endothelial cells or in cells placed very close to the endothelium. Identical Snail1 expression levels were observed for control mice (Snail1^Flox^/^+^) after TAM injection or in Snail1^Flox^/^−^ mice prior to TAM treatment (Figure 3). No PMCs positive for Snail1 were observed in Snail1^Flox^/^−^ mice one week after TAM injection (Figure 3).

**Figure 4:**
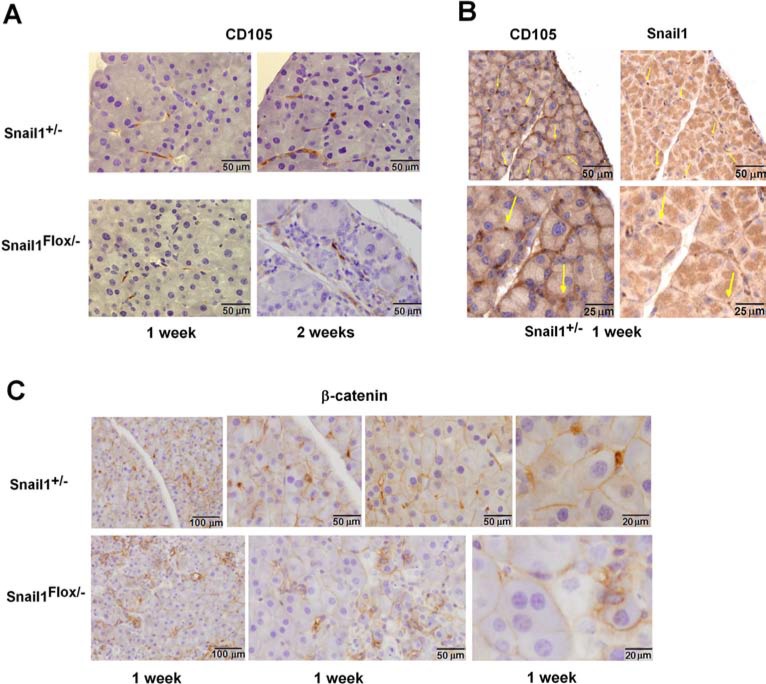
Nuclear β-catenin is absent from PMCs in Snail1-deficient mice Expression of CD105, (**A, B**) or β-catenin (**C**) was determined as indicated in the pancreata from control or Snail1-deficient mice one or two weeks after treatment with tamoxifen. In (B) consecutive sections were stained for Snail1 and CD105; cells showing expression of both markers are labeled with arrows. The bars indicate magnification.

We also stained pancreata with antibodies corresponding to other mesenchymal markers. In control mice, CD105 presents a similar distribution as Snail1 in the pancreas (Figure 4A–4B) and was detected in fibroblastic cells close to or surrounding the acini; in fact, many Snail1-positive cells also expressed CD105 (Figure 4B). However, CD105 showed a broader distribution than Snail1. Pancreatic expression of CD105 was maintained in Snail1-depleted animals: its expression pattern did not significantly change one week after TAM administration but differed after two weeks, likely reflecting the fact that the pancreatic architecture was severely compromised by this time. Other mesenchymal markers, such as desmin, S100A4, and glial fibrillary acidic protein (GFAP), were also present in PMCs, although they showed a different pattern than Snail1, suggesting that they were present in a different set of mesenchymal cells (Supplementary Figure S8). These markers were also expressed in Snail1-depleted mice.

Since Snail1 controls β-catenin distribution (See Introduction), we also determined β-catenin subcellular localization in the pancreas. In addition to a general staining in the cell periphery in epithelial cells, a strong β-catenin nuclear accumulation was observed in cells with an elongated phenotype located close to the acini, which resembled those positive for Snail1 (Figure 4C). Although β-catenin expression in epithelial cells was maintained in Snail1-depleted pancreata, cells with nuclear β-catenin were no longer detected. These results suggest that, similar to fibroblasts [6, 7], PMCs require Snail1 for β-catenin nuclear distribution.

### Depletion of Snail1 in cultured PMCs decreases expression of β-catenin targets and alters the PMCs secretome

**Figure 5:**
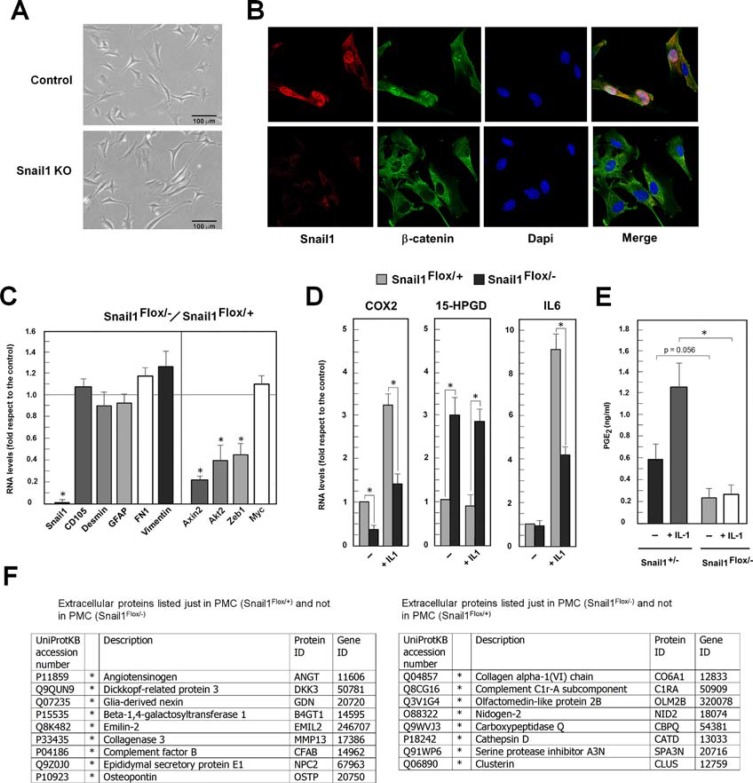
Snail1-deficient PMCs present decreased nuclear β-catenin and downregulated expression of β-catenin–target genes PMCs were isolated from Snail1^Flox/–^ mice and purified by FACS sorting with anti-CD105 antibodies. Cells were infected with a retrovirus expressing the Cre recombinase or with a control virus, selected and analyzed. A micrograph of control or Snail1-deficient cells is shown in panel (**A**) an immunofluorescence analysis with the indicated antibodies, in (**B**) and the analysis of the RNA corresponding to the indicated genes, in (**C–D**). When indicated, cells were starved in 2% serum overnight and treated with IL1α (1 ng/ml) for 48 hr. The average (± SD) of the results of three experiments is shown. In (**E**) the levels of PGE_2_ were determined in the culture medium of control or Snail1-deficient cells upon incubation with IL1α when indicated. The asterisk indicates that the differences are significant with *p* < 0.05. (**F**) PMCs either control or Snail1-deficient were cultured and their conditional medium was obtained and analyzed as indicated in Supplementary Methods. The table shows the proteins categorized as Extracellular Proteins that were detected specifically in one of the two cell populations. The differential expression of these proteins was validated in another analysis of independent samples

To study the role of Snail1 in PMCs, we isolated and cultured these cells. However, primary PMC cultures showed a heterogeneous composition; so, we took advantage of the fact that CD105 is expressed in Snail1-positive cells, and the availability of CD105 antibodies suitable for FACS selection, to further purify these cells. Indeed, CD105-purified PMCs presented a homogeneous phenotype and expressed Snail1 (Figure 5A–5B); Snail1 depletion by Cre expression neither modified their morphology nor affected their proliferation.

As in other cultured mesenchymal cells [8], Snail1 was mainly present in the nucleus, co-localizing with β-catenin (Figure 5B). Snail1 depletion promoted the exclusion of β-catenin from the nucleus (Figure 5B), in agreement with previously findings that β-catenin is a transcriptional element that is translocated to the nucleus by Snail1 [6]. We did not detect a significant decrease in the expression of other mesenchymal markers such as desmin, GFAP, CD105, fibronectin, vimentin, or S100A4 upon Snail1 depletion (Figure 5C). However, the expression of targets of the transcriptional activity of the β-catenin/Tcf-4 complex, such as axin2 and others (Zeb1, Akt2, Cox2) [20, 21], was downregulated in Snail1 KO PMCs (Figure 5C–5D). Cox2 stimulation by interleukin 1α (IL1 α) [22] was also lower in the absence of Snail1 (Figure 5D).

Besides Cox2, Snail1 has been shown to control another enzyme involved in prostaglandin metabolism, 15-Hpgd [23]; therefore we also checked the RNA levels corresponding to this gene. 15-Hpgd RNA was clearly upregulated in Snail1-deficient PMCs with respect to the control (Figure 5D). Probably as consequence of Snail1's effects both on Cox2, which is required for PGE_2_ synthesis, and on 15-Hpgd, which degrades this prostaglandin, the levels of PGE_2_ were lower and were not stimulated by IL1 α in Snail1-deficient PMCs, in contrast to control cells (Figure 5E).

We also analyzed the proteins differently secreted by PMCs, either control or Snail1-deficient. Cells were grown and conditional medium was obtained, concentrated and analyzed. We detected 32 and 34 proteins exclusively present in the conditional medium from PMCs control or Snail1-deficient, respectively. The lists of these proteins are included in Supplementary Tables S1 and S2 and those categorized as Extracellular Proteins in Figure 5F. Several of the proteins secreted by control cells and absent from Snail1-KO PMCs correspond to proteins (DKK3, MMP13, OSTP, S10A6) involved in the activation of fibroblasts [24–27] further suggesting that elimination of Snail1 prevents this process.

### Snail1 expression in PMCs is required to maintain the acinar characteristics in cultured pancreas cells

**Figure 6:**
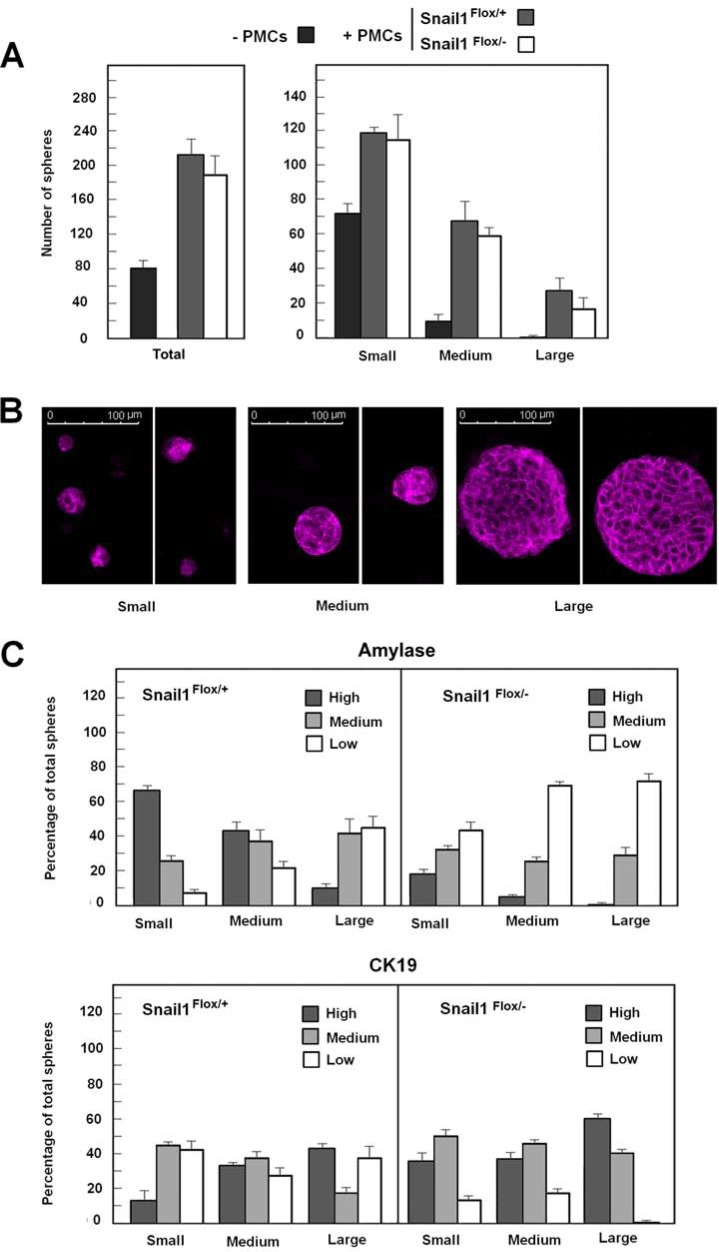
Snail1 expression in co-cultured PMCs retards loss of acinar markers in pancreas cells Pancreas explants were prepared as described in Materials and Methods and cultured in the absence or presence of PMCs (either wild-type or KO for Snail1). The number of spheres and their relative size was determined (panel **A**). Representative examples of large, medium, and small spheres stained with E-cadherin are shown in panel (**B**). Spheres were also stained with amylase- or CK19-specific antibodies as indicated (see also Supplementary Figure S7), and the expression of these markers was categorized as high, medium, or low. The percentage of the spheres showing the different levels of staining is presented in panel (**C**). The figure shows the average (± range) from two experiments

When acinar cells are cultured, acinar markers are rapidly lost while ductal ones are upregulated in a process named acinar-ductal metaplasia. To determine whether PMCs affect this process, we cultured pancreas cells in a medium with Matrigel (See Materials and Methods); under these conditions, we detected the formation of spheres comprising epithelial cells. The number of these epithelial spheres was much higher when acinar explants were supplemented with PMCs (Figure 6A, left panel). These PMCs grew in culture without adhering to the spheres (Supplementary Figure S9). The total number of these structures did not differ irrespective of whether acinar cells were co-cultured with wild-type or Snail1-deficient PMCs. By classifying these epithelial spheres according their size, we found that large spheres (with a diameter longer than 80 μm) presented a much more defined E-cadherin localization than medium or small ones (Figure 6B). These large spheres were not detected when cultures were not supplemented with PMCs and were slightly more abundant when PMCs expressed Snail1 (Figure 6A, right panel).

We also analyzed the expression levels of acinar (amylase) or ductal (CK19) markers, categorized as high, medium, or low; representative examples are presented in Supplementary Figure S10. Amylase expression was higher in small than in large spheres, whereas CK19 was enriched in large spheres (Figure 6C). Further, 65% of the small structures expressed high levels of amylase, but only 19% had high CK19; conversely, only 11% of the larger spheres expressed high levels of amylase whereas 47% had high CK19. Snail1 depletion in PMCs increased CK19 and downregulated amylase expression in all these structures; for instance, the percentage of amylase-high structures decreased in Snail1 KO PMCs as compared to wild-type PMCs, from 65 to 19, 38 to 5, and 11 to 0 in small, medium, or large structures, respectively (Figure 6C). On the contrary, CK19 expression was upregulated in Snail1 KO PMCs, and the percentage of CK19-rich spheres increased from 19 to 40, 34 to 39, and 43 to 61 for small, medium and large spheres, respectively. Although these results were not repeated enough times to provide statistical significance, they suggest that the presence of Snail1 in PMCs contributes to a better maintenance of acinar markers in cultured pancreas cells.

### Snail1 ablation affects the phenotype of pancreas tumors

**Figure 7:**
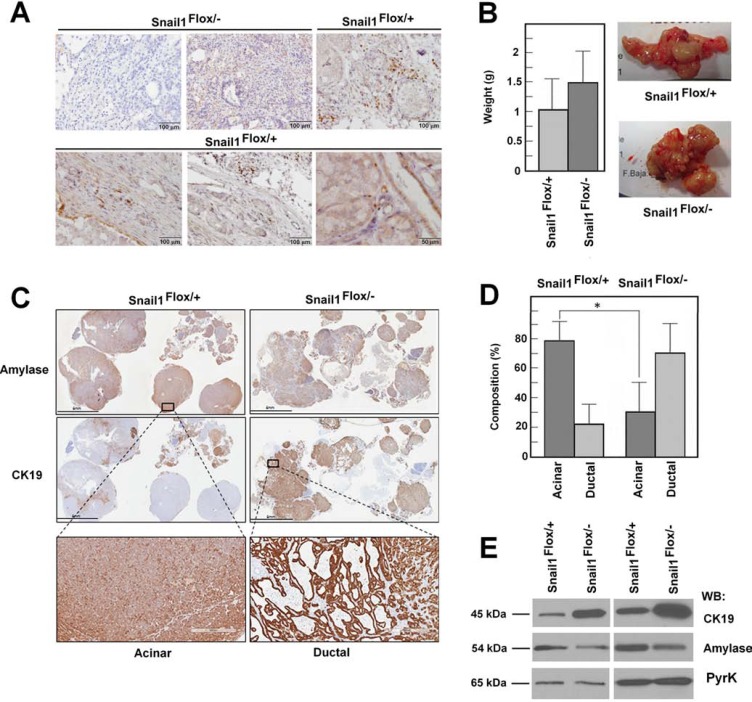
Snail1 is expressed in the stroma of Ela-myc tumors and contributes to maintaining the acinar phenotype Snail1 depletion was carried out by TAM injection in 2.5-month-old mice; tumor size and characteristics were studied one month later. expression of Snail1 was assessed in panel (**A**), tumor size, in (**B**), and the acinar or ductal composition of the tumors, in panels (**C** and **D**), An acinar or ductal phenotype was determined on the basis of the amylase and CK19 expression and by the phenotype of the tumors. A representative staining of the entire tumor is presented in C, with enlarged areas corresponding to acinar or ductal compartments. The average of the percentage of acinar and ductal composition at tumors generated in control and Snail1-deficient mice is shown in panel D. Total protein from two representative tumors from each control or Snail1-deficient mice were analyzed by Western blot using antibodies against CK19, amylase, or PyrK as loading control (**E**). The asterisk indicates that the differences are significant with *p* < 0.05.

Finally, we also determined if Snail1 expression affects the development of pancreas tumors using the Ela- Myc transgenic mice. This murine line expresses the c-Myc proto-oncogene under the control of the Elastase promoter, which is specific for acinar cells, and animals develop tumors with a high penetrance between 2 and 5 months of age [28]. In the initial phase (2–3 months), these tumors present extensive areas of acinar cell carcinomas, and they progress to ductal adenocarcinomas at later times [29]. Generation of ductal tumors is preceded by acinar-ductal metaplasia, a transdifferentiation process that also likely triggers pancreatic adenocarcinoma in humans [30–31]. We induced Snail1 depletion when mice were 2.5 months old; animals were euthanized one month after TAM administration, and tumor size and morphology was evaluated. Pancreas tumors presented Snail1 expression; this factor was detected in the nuclei of mesenchymal cells located in the stroma in areas of desmoplasia or embracing acinar structures (Figure 7A). A similar stromal distribution of Snail1 was observed in a small collection of human pancreas neoplasic and preneoplasic lessions (Supplementary Figure S11). Only stromal cells present in desmoplastic areas with fibroblastic infiltration expressed Snail1.

In contrast, tumors from Snail1-deficient mice did not show expression of this factor; only in some cases a low unspecific staining in the cytosol was observed (Figure 7A). Depletion of Snail1 did not significantly modified tumor weight (Figure 7B). However, the cellular characteristics of the tumors were different, and the proportion of mature acinar cells in the tumors from Snail1-depleted animals was remarkably lower than in those from wild-type animals. Acinar or ductal components were determined by analyzing the expression of amylase and CK19 markers, specific for acinar or ductal cells, respectively (Figure 7C), and on the basis of the cellular phenotype, determined after eosin-hematoxylin staining. Acinar hyperplasia was included in the “acinar phenotype,” whereas ductal phenotype at this stage was mainly acinar-ductal metaplasia and occasionally differentiated ductal adenocarcinomas. The percentage of tumors with acinar characteristics was high in control tumors at 3.5 months; in contrast, the predominant tumor phenotype in Snail1-deficient mice was ductal (acinar-ductal metaplasia or ductal adenocarcinoma) (Figure 7D). Accordingly, amylase was enriched in tumors derived from control versus Snail1 KO animals, whereas tumors obtained from these mice presented higher levels of CK19 (Figure 7E). Therefore, and in accordance with our previous findings indicating that Snail1 is required to maintain the pancreas acini, Snail1 depletion favors the progression from acinar to ductal adenocarcinomas by increasing acinar to ductal metaplasia.

## DISCUSSION

We show here that Snail1 expression in pancreatic mesenchymal cells is necessary to maintain a normal pancreas structure. Snail1 depletion causes the rapid loss of acinar cells and their substitution by adipocytes. A similar replacement of acinar cells by adipocytes has been observed in other transgenic models, although at longer times. For instance, inhibition of TGF-β signaling in acinar cells by constitutive expression of a dominant-negative mutant of the TGF-β receptor induces acini loss, appearance of ductular structures, and progressive replacement by adipocytes that become predominant by five months of age [32]. Loss of acinar cells and accumulation of adipocytes in aged animals (10 months) have also been observed in mice with a c-myc inactivation in Pdx-1–positive pancreas progenitor cells [33]. Lineage tracing experiments performed in this model indicated that adipocyte cells were directly derived from the transdifferentiation of acinar cells. A drastic change in pancreas size and the appearance of fat cells have been described following inactivation of the Hippo pathway [34]. A less severe phenotype has also been observed in mice with IKKα ablation in the epithelial cells from the pancreas [35]. These animals suffer a loss of acinar cells accompanied by characteristic features of pancreatitis, such as inflammation and circulatory release of pancreatic enzymes.

In our animal model, loss of Snail1 induces a rapid and significant apoptosis of acinar cells that precede the adipocytes onset. It is unlikely that these cells are generated from the pancreatic stellate cells (PSCs), since adipocytes seem to be too abundant; however, it is possible that they could come from fibroblasts recruited to the damaged pancreas. Alternatively, they might stem from the transdifferentiation of a group of acinar cells resistant to apoptosis that undergo this process in the absence of the paracrine signals from PMCs. The internal or external cues that control whether an acinar cell undergoes apoptosis or transdifferentiation are still unknown.

Our model has significant differences to other transgenic mice that accumulate adipocytes in the pancreas. For instance, previous models were mostly based on the constitutive inactivation of a target gene using a Cre recombinase under the control of the Pdx- 1 promoter, which is active in pancreas progenitor cells during development. In our assays, Snail1 depletion was performed in adult animals, and the appearance of the phenotype was remarkably accelerated with respect to other models, as adipocytic cells accumulated one month after Snail1 depletion. Further, in our model, acinar cells were lost quickly, whereas ducts were conserved; in fact, at four weeks, few acinar cells were present, and most of remaining epithelial cells were ductal cells positive for CK19. Moreover, although we observed acinar loss and fibroblast accumulation, we did not detect other classical features of pancreatitis, such as a systemic increase in digestive enzymes. Also, in agreement with other mice models in which acini were replaced by adipocytes, islets were not compromised by Snail1 depletion and did not show any alteration in their structure or function.

Snail1-positive mesenchymal cells were located mostly embracing the acini in control pancreas. Among the mesenchymal markers analyzed, Snail1 expression resembled that of CD105, enabling us to use antibodies against this protein to isolate and enrich mesenchymal cells. Depletion of Snail1 did not cause a significant change in the phenotype or proliferation of these cells; however, it selectively affected the expression of specific genes, such as Cox2, either in basal conditions or upon its activation by IL1α. Besides Cox2, the expression of other β-catenin-targets genes, such as Axin2 or Zeb1, was also diminished following Snail1 depletion. This is likely due to the altered distribution of β-catenin in PMCs, which is absent from the nuclei in Snail1-deficient cells. Several reports have already described that the transcriptional activity of β-catenin is stimulated by Snail1, at least in part through a mechanism involving direct interaction [7]. Probably as the consequence of a lower expression of Cox2 and a higher by “expression of 15-Hpgd (which is involved in PGE_2_ catabolism), Snail1 KO cells responded to IL-1α with a much lower stimulation of PGE_2_ secretion than the control cells.

The best characterized mesenchymal cells in the pancreas are the PSCs [36]. In normal pancreas, these cells are considered to be quiescent and express very few specific markers; however, following pancreatic damage, they are turned on and activate proteins such as smooth muscle actin (SMA). We have not been able to determine if the mesenchymal cells expressing Snail1 correspond to PSCs, since they do not co-stain with most putative PSC markers, such as SMA, S100A4, and GFAP. However, it is possible that Snail1-positive cells correspond to a subset of PSCs, especially considering that a role for resting PSCs in pancreas architecture has been suggested [33]. PSC-specific depletion in β1 integrin decreases the extracellular matrix expression and affects acinar cell interactions with basement membrane; these alterations are accompanied with lower zymogen production, apoptosis, and a partial decrease in pancreas size [37, 38]. The effects observed by these authors are milder than those produced by Snail1 depletion, which are likely to not be limited to modifications in extracellular matrix proteins. Moreover, β1 integrin depletion does not cause adipocytes to accumulate in the pancreas. It is possible that, as described in other mesenchymal cells [12], Snail1 is required for β1 integrin expression in PSCs and only labels (or marks) these cells at an initial phase of activation. In any case, all these studies indicate a fundamental role of PMCs in pancreas homeostasis.

PMCs lacking Snail1 are also less capable of maintaining an acinar cell phenotype when these cells are cultured *ex vivo*. Primary pancreas cells are capable of forming large spheres when grown in Matrigel. The number and especially the size of these spheres greatly depend on supplementing the culture with PMCs. The expression of Snail1 in these cells does not affect their number and only slightly affects their size, but it does modify the composition of these structures. Without Snail1 in PMCs, the loss of acinar markers happens earlier and especially the large spheres are totally devoid of acinar markers, such as amylase. This result suggests again that depletion of Snail1 in PMCs prevents the secretion by these cells of extracellular factors required for acinar survival.

The results obtained in the animal tumor model also reinforce the relevance of Snail1 in acinar maintenance. Myc over-expression in acinar cells generates pancreas carcinomas that express Snail1 in the stroma; these lesions display acinar-like and duct-like neoplastic cells. The ratio of these two cell types depends on tumor time of life, since the initial neoplasms present a higher proportion of acinar areas, whereas the later ones are enriched in duct-like structures due to acinar-ductal metaplasia [28]. Our results indicate that this process of metaplasia is negatively regulated by Snail1, since it is accelerated in mice deficient for the expression of this gene, without a significant alteration in tumor size. Therefore, PMCs expressing Snail1 are relevant for maintaining acinar phenotype not only in healthy animals but also in neoplasias.

Ectopic Snail1 expression in acinar cells of another murine model of pancreas cancer (Kras ^G12D^) accelerates acinar-ductal metaplasia [39]. This mutated Kras model differs from ours since it requires additional mutations to progress to a ductal adenocarcinoma [40–41]. Snail1 depletion in Pdx-positive cells does not inhibit pancreatic tumorigenesis in the K-ras^G12D^, p53^R172H^ transgenic mouse model, neither it prevents tumor metastasis [42], suggesting that Snail1 expression in epithelial cells is not relevant for the progression of this neoplasia. Indeed, our preliminary results analyzing Snail1 expression in pancreas tumors indicate that it is present specifically in the tumor stroma (Supplementary Figure S11). It remains to be established whether Snail1 action in tumors is similar to that performed in normal tissue or besides helping to maintain acinar cells its expression in the tumor stroma also favors progression, as it has been suggested in other epithelial neoplasms [14, 43].

## MATERIALS AND METHODS

### Mice

The generation of a conditional knock-down Snail1 mouse carrying null [13] and the Snail1^flox^ conditional allele [44] has been described [12]. This mouse also holds a Cre recombinase–estrogen receptor fusion gene under the control of β-actin promoter (β-actin Cre-ER). Animals that were β-actin–CreER, Snail1 null (Snail1^−^), Snail1 ^flox^ (Snail1^Flox/–^) were used for further analysis. Animals bearing one copy of the wild-type Snail1 gene (Snail1^Flox/+^) were used as controls. Another murine line was obtained by mating Snail1-null and Snail1-floxed alleles with mice holding Cre-ER recombinase under the control of PTF1/p48 promoter. These animals were obtained from The Jackson Laboratory. Activation of Cre recombinase was induced in 2− to 2.5-month-old animals by three peritoneal injections of tamoxifen (0.1 mg per g of mouse weight, dissolved in corn oil) at alternative days. Ela-Myc animals were obtained from E. Sandgren (Univ of Wisconsin, USA) and bred to obtain β-actin–CreER, Snail1^flox^, Snail1^−^ (or Snail1+), Ela-myc animals. Mice were housed and regularly followed according to procedures established and approved by the Animal Research Ethical Committee (CEEA) of the PRBB. Ela-myc transgenic mice were sacrificed at 3.5 months of age, or one month after tamoxifen injection when appropriate; an autopsy was performed, and the pancreas was resected and processed for histological analysis [45]. All mice involved in this study were maintained in a rodent barrier facility to guarantee the specific pathogen free (SPF) health status of the animals. All animal experiments were previously approved by the Animal Research Ethical Committee from the PRBB. Analysis of murine sera was performed according to standard procedures at Laboratorios Echevarne (Barcelona, Spain). Statistical analyses were carried out using SPSS 18.0; *p* values lower than 0.05 are symbolized by one asterisks, lower tan 0.01, by two asterisks; other *p* values between 0.1 and 0.05 are indicated.

### Pancreatic mesenchymal cell isolation and culture

Pancreatic mesenchymal cells (PMCs) were isolated by a modification of the method described [46]. Briefly, pancreatic tissue from 5 mice was pooled, minced with scissors, and digested with 0.02% pronase, 0.05% collagenase P, and 0.1% DNAse in Gey's balanced salt solution (GBSS), for 15 min at 37°C. Digested tissue was pipetted through successively narrower orifices and then filtered through a 150 μm nylon mesh. Cells were washed and resuspended in 1.9 ml GBSS containing 0.3% BSA. The cell suspension was mixed with 1.6 ml of 28.7% (wt/vol) of Nycodenz in Gey's solution without salt. The Nycodenz gradient was prepared by layering the cell suspension in Nycodenz underneath 1.2 ml Gey's solution with BSA in a 10 ml centrifuge tube. The gradient was centrifuged for 20 minutes at 1400 × g. The cells of interest separated into a fuzzy band just above the interface of the Nycodenz cushion and the GBSS with BSA. This band was harvested, and the cells were washed, resuspended in Iscove's modified Dulbecco's medium containing 10% fetal bovine serum (FBS), 4 mM glutamine, and antibiotics (penicillin 100 units/ml; streptomycin 100 μg/ml), and cultured in the same medium. PMCs were selected by FACS using an antibody against CD105 (Abgent, 1/30) and a secondary antibody labeled with Alexa 488 (1/200). Sorted cells were infected with a retrovirus expressing the Cre recombinase or with a control virus, selected with puromycin (1 μg/ml), and analyzed.

### Collection and culture of acinar pancreas cells

After mice sacrifice, pancreas was injected with 2.5 ml of collagenase (1.33 mg/ml), dissected, and incubated with 5 ml of collagenase for 20 min at 37°C. The reaction was stopped by adding 10 ml of Hanks' Balanced Salt Solution (HBSS) (Gibco) supplemented with FBS (5%) and centrifuged at 200 × g for 2 min. The pellet was resuspended in HBSS plus FBS, and the process of centrifugation was repeated two more times. Finally, the resuspended cellular pellets were filtered twice, first through a 500 μm polypropylene mesh (Spectrum Labs, ref 146418) and then though a 100 μm nylon mesh (Spectrum Labs, ref 146488). The final suspension (10 ml) was added on top of 20 ml of HBSS+ 30% FBS and centrifuged as above. Pelleted cells were resuspended in 20 ml of RPMI (supplemented with 10% FBS and 25 μg/ml of G418 used to eliminate endogenous fibroblast), and cells proceeding from one pancreas were divided in two and mixed with 7 × 10^5^ PMCs, either wild-type or Snail1 KO, previously transfected with pIRES to confer G418 resistance. Cell mixes were incubated for 1 hr at 37°C before culture.

Cells were split 1/6, pelleted down at 400 × g for 4 min, and carefully resuspended in 250 μl of cold Matrigel (Biosciences BD). A 50 μl drop was seeded in a well and incubated for 15 min at 37°C; 450 μl of RPMI medium plus FBS and G418 was added, and cells were incubated at 37°C, changing the medium every 48 hr. Samples were examined at day 3 to determine the number and size of spheres. Size was determined as large (diameter longer than 80 μm), medium (between 40 and 80 μm), or small (shorter than 40 μm). The cellular structures were also analyzed by immunofluorescence with antibodies against E-cadherin, CK19, and amylase. For this, Matrigel was removed by incubation with Corning Cell Recovery Solution (Cat n° 354253) at 4° for 1 h. Cells were pelleted down as before, resuspended in p-formaldehyde (PFA) (4%), and incubated for 15 min. Immunofluorescence analysis was performed as described below. Staining was scored on a scale of 0 to 300, the result of multiply percentage of positive cells and intensity of immunoreactivity. Expression in these samples was categorized into three groups: low (< 10), medium (between 10 and 100), and high (> 100).

### Pancreas extract preparation and western blot analysis

To prepare pancreas extracts, mice were euthanized, and pancreata (normal or pathological) were removed and frozen in liquid N_2_. Pancreata were lysed in buffer L1 (50 mM Tris-HCl pH 8.0, 2% SDS, 10% glycerol) plus phosphatase (1 mM β-glycerol phosphate, 10 mM NaF, 1 mM sodium orthovanadate) and protease inhibitors (complete mini cocktail, ROCHE), using Lysing Matrix D (MP #6913–050). Lysate was heated at 95°C for 5 min, passed sequentially through 18G, 21G, 23G, and 25G syringes, sonicated for 10 min, and centrifuged at 13, 600 × g for 10 min. Protein (5 μg) was fractionated by 10% or 15% SDS-PAGE and analyzed by Western Blot using the following antibodies: anti-amylase (Sigma-Aldrich), anti-chymotrypsinogen (Biogenesis), anti-β-actin (Abcam), or anti–pyruvate kinase (Merck).

### RNA extraction and analysis

RNA was extracted with TRizol using standard procedures. Expression levels of transcripts were also calculated by real-time PCR, using the Transcriptor First Strand cDNA Synthesis kit (Roche) and the LightCycler^®^ 480 Real-Time PCR System (Roche). RNA levels were determined quantitatively in triplicate on a LightCycler 480 system. The relative quantification value for each target gene as compared with the calibrator for that target is expressed as 2^−(Ct-Cc)^ (where Ct and Cc are the mean threshold cycle differences after normalizing to HPRT expression). Reactions were performed according to the manufacturer's directions, using the primers presented in Supplementary Table S3.

### Immunofluorescence analysis of PMCs and acinar cultured cells

PMCs were grown on gelatin-treated coverslips and fixed with PFA (4%) for 15 min. After permeabilization in 1% Tween-20 and blocking, cells were incubated for 1 hr with rabbit anti–β-catenin (SIGMA, C2206) and mouse monoclonal anti-Snail1 (8). After washing, samples were incubated with secondary antibodies anti-murine IgGs (labelled with Alexa 555) or anti-rabbit IgGs (labelled with Alexa 647 (1/500) (all from Invitrogen). Cells were mounted with Flouromount G–DAPI to counterstain nuclei and visualized in a Leica SPE confocal microscope.

Matrigel-released cultured cells were fixed with PFA (4%) and extended on a coverslip after a quick spin (30 sec, 400 × g). They were fixed again with PFA for 5 min, washed, permeabilized in 0.3% Triton *X*-100, and incubated with antibodies against E-cadherin (Transduction Labs), amylase (Sigma-Aldrich), or CK19 (Abcam). After washing, samples were incubated with secondary antibodies labelled with Alexa 488, Alexa 555, or Alexa 647 (Invitrogen) and analysed in the confocal microscope.

### Immunohistochemical analysis of pancreas

Immunohistochemical analysis of Snail1 protein in normal and neoplasic pancreas was performed as previously described by using mAb EC3 [8, 14], using 4 μm sections. For antigen unmasking, sections were immersed in Tris-EDTA buffer (pH 9) and boiled for 20 min. Immunohistochemical staining was carried out with anti-Snail1 MAb EC3 supernatant at 1/300 (murine) or 1/100 (human) dilution using the CSAII Amplification System (Dako, Glostrup, Denmark), in a Dako Link platform. Human samples (six PanIN and five pancreas adenocarcinomas) were obtained from Parc de Salut MAR Biobank (MARBiobanc), Barcelona. The analyses were approved by the Ethical Committee for Clinical Research of PRBB (Barcelona) and informed consent was obtained from all subjects.

Other proteins were analyzed with the following antibodies: desmin, GFAP (both from DakoCytomation), β-catenin (Sigma Aldrich or Transduction Labs), amylase (Sigma-Aldrich), CK19 (Abcam), CD105 (Abgent), S100A4 (Millipore), insulin, glucagon, and active caspase-3 (Cell Signalling). Sections were counterstained with hematoxylin.

## SUPPLEMENTARY MATERIALS TABLES AND FIGURES


