# Supplementary material for: Snail1 is required for the maintenance of the pancreatic acinar phenotype
Source: Oncotarget. 2015 Dec 29;7(4):4468–82. doi: 10.18632/oncotarget.6785 (PMC4826219; doi:10.18632/oncotarget.6785)
Supplement: Supplementary file 1 [file oncotarget-07-4468-s001.pdf]

Snail1 is required for the maintenance of the pancreatic acinar phenotype

Supplementary Materials

MATERIALS AND METHODS

Immunofluorescent analysis of pancreas

Double immunoHistoFluorescence analysis of Insulin and Glucagon on paraffin sections was performed as follows: after antigen retrieval by boiling the slides in citrate buffer pH 6 buffer during 10 min, samples were blocked and incubated overnight at 4°C with anti-Glucagon rabbit antiserum (Santa Cruz sc13091) and anti-Insulin monoclonal antibody (SIGMA I2018). After a washing, samples were incubated with anti-rabbit Alexa 488 and anti-mouse Alexa 555, counterstained with DAPI and mounted with Flouromount.

The triple analysis of Active Caspase 3, Amylase and CK19 was performed with rabbit anti Active Caspase 3 (Cell Signalling 9661), goat anti CK19 (Santa Cruz, sc-33111) and murine anti Amylase (Santa Cruz, sc-49957) all of them were used at 1/50 in PBS plus BSA (2%) for 1 h at room temperature. Anti-mouse Alexa 488, anti-rabbit Alexa 555 and anti-goat Alexa 647 conjugated fluorescent secondary antibodies were used for 1h at room temperature 1/500 in PBS plus BSA (2%). After DAPI staining, samples were mounted in Flouromount and images were captured on Leica SPE confocal microscope.

Secretome analysis

Wild-type or Snail1-depleted PMCs (106) cells were plated in a non-gelatine coated T150 flask in IMD medium with 10% FBS. After 24 h, cells were washed twice with PBS and cultured overnight with 4 ml of IMD medium without FBS. Sample media was concentrated by ultrafiltration with a MWCO membrane (Vivaspin 6, 3000 MW; Sartorius, Gottingen, Germany) and precipitated by the addition of six volumes of acetone (overnight, 4°C). Precipitated proteins were then dissolved in 50 µl of 6 M Urea plus 200 mM NH4HCO and protein content was determined using the BCA Protein Quantification Kit (Thermo Fisher Scientific). Samples were reduced with dithiothreitol (60 nmol, 1 h, 37°C) and alkylated in the dark with iodoacetamide (120 nmol, 30 min, 25°C). The resulting protein extract was first diluted 1/3 with 200 mM NH4HCO3 and digested with 0.2 µg of LysC (Wako, cat # 129–02541) overnight at 37°C and then diluted 1/2 and digested with 0.2 µg of trypsin (Promega, cat # V5113) for eight hours at 37°C. Finally, the peptide mix was acidified with formic acid and desalted with MicroSpin C18 column (The Nest Group, Inc) prior to LC-MS/MS analysis.

Samples (1 mg) were analyzed using a LTQ-Orbitrap XL mass spectrometer (Thermo Fisher Scientific, San Jose, CA, USA) coupled to an EasyLC (Thermo Fisher Scientific (Proxeon), Odense, Denmark). Peptides were loaded directly onto the analytical column at a flow rate of 1.5–2 µl/min using a wash-volume of 4 times the injection volume, and were separated by reversed-phase chromatography using a 25–cm column with an inner diameter of 75 µm, packed with 5 µm C18 particles (Nikkyo Technos Co., Ltd. Japan). Chromatographic gradients started at 93% buffer A and 7% buffer B for 7 minutes with a flow rate of 250 nl/min, and gradually increased to 65% buffer A and 35% buffer B in 60 min. After each analysis, the column was washed for 15 min with 10% buffer A and 90% buffer B. Buffer A: 0.1% formic acid in water. Buffer B: 0.1% formic acid in acetonitrile.

The mass spectrometer was operated in positive ionization mode with nanospray voltage set at 2.5 kV and source temperature at 200°C. Ultramark 1621 for the FT mass analyzer was used for external calibration prior the analyses. Moreover, an internal calibration was also performed using the background polysiloxane ion signal at m/z 445.1200. The instrument was operated in DDA mode and full MS scans with 1 micro scans at resolution of 60,000 were used over a mass range of m/z 350–1500 with detection in the Orbitrap. Auto gain control (AGC) was set to 1E6, and dynamic exclusion (60 seconds) and charge state filtering disqualifying singly charged peptides were both activated. In each cycle of DDA analysis, following each survey scan the top twelve most intense ions with multiple charged ions above a threshold ion count of 5000 were selected for fragmentation at normalized collision energy of 35%. Fragment ion spectra produced via collision-induced dissociation (CID) were acquired in the ion trap, AGC was set to 5E4, isolation window of 2.0 m/z and maximum injection time of 50 ms was used. All data were acquired with Xcalibur software v2.2.

Data analysis

Proteome Discoverer software suite (v1.4, Thermo Fisher Scientific) and the Mascot search engine (v2.5, Matrix Science) were used for peptide identification and quantification. Samples were searched against against a SwissProt database containing entries corresponding to Mouse (version of May 2015) a list of common

contaminants and all the corresponding decoy entries. Trypsin was chosen as enzyme and a maximum of three miscleavages were allowed. Carbamidomethylation (C) was set as a fixed modification, whereas oxidation (M) and acetylation (N-terminal) were used as variable modifications. Searches were performed using a peptide tolerance of 7 ppm, a product ion tolerance of 0.5 Da. Resulting data files were filtered for FDR < 5 %.

The proteomics analyses were performed in the "CRG/UPF Proteomics Unit, Centre de Regulació Genòmica (CRG), Universitat Pompeu Fabra (UPF), 08003 Barcelona". The CRG/UPF Proteomics Unit is part of the Spanish Platform of Molecular and Bioinformatics Resources (ProteoRed), Instituto de Salud Carlos III (PT13/0001).

Supplementary Table S1: Proteins listed just in PMC (Snail1Flox/+) and not in PMC (Snail1Flox/-)

UniProtKB accession number		Description	Protein ID	Gene ID
P11859	*	Angiotensinogen	ANGT	11606
Q9QUN9	*	Dickkopf-related protein 3	DKK3	50781
Q07235	*	Glia-derived nexin	GDN	20720
P15535	*	Beta-1, 4-galactosyltransferase 1	B4GT1	14595
Q8K482	*	Emilin-2	EMIL2	246707
P33435	*	Collagenase 3	MMP13	17386
P04186	*	Complement factor B	CFAB	14962
Q9Z0J0	*	Epididymal secretory protein E1	NPC2	67963
P10923	*	Osteopontin	OSTP	20750
Q8R550		SH3 domain-containing kinase-binding protein 1	SH3K1	58194
P47757		F-actin-capping protein subunit beta	CAPZB	12345
P14069		Protein S100-A6	S10A6	20200
O88544		COP9 signalosome complex subunit 4	CSN4	26891
Q8BP47		Asparagine—tRNA ligase, cytoplasmic	SYNC	70223
Q9Z1Z2		Serine-threonine kinase receptor-associated protein	STRAP	20901
P46061		Ran GTPase-activating protein 1	RAGP1	19387
P63087		Serine/threonine-protein phosphatase PP1-gamma catalytic subunit	PP1G	19047
Q9CQC6		Basic leucine zipper and W2 domain-containing protein 1	BZW1	66882
Q64213		Splicing factor 1	SF01	22668
P62918		60S ribosomal protein L8	RL8	26961
Q9CZ30		Obg-like ATPase 1	OLA1	67059
Q99LP6		GrpE protein homolog 1, mitochondrial	GRPE1	17713
P56480		ATP synthase subunit beta, mitochondrial	ATPB	11947
Q8R081		Heterogeneous nuclear ribonucleoprotein L	HNRPL	15388
P38647		Stress-70 protein, mitochondrial	GRP75	15526
Q9JMK0		Beta-1, 4-galactosyltransferase 5	B4GT5	56336
P62270		40S ribosomal protein S18	RS18	20084
P62908		40S ribosomal protein S3	RS3	27050
Q60668		Heterogeneous nuclear ribonucleoprotein D0	HNRPD	11991
P15864		Histone H1.2	H12	50708
Q61079		Single-minded homolog 2	SIM2	20465
P97493		Thioredoxin, mitochondrial	THIOM	56551

*extracellular

Supplementary Table S2: Proteins listed just in PMC (Snail1$^{Flox/-}$) and not in PMC (Snail1$^{Flox/+}$)

UniProtKB accession number		Description	Protein ID	Gene ID
Q04857	*	Collagen alpha-1(VI) chain	CO6A1	12833
Q8CG16	*	Complement C1r-A subcomponent	C1RA	50909
Q3V1G4	*	Olfactomedin-like protein 2B	OLM2B	320078
O88322	*	Nidogen-2	NID2	18074
Q9WVJ3	*	Carboxypeptidase Q	CBPQ	54381
P18242	*	Cathepsin D	CATD	13033
Q91WP6	*	Serine protease inhibitor A3N	SPA3N	20716
Q06890	*	Clusterin	CLUS	12759
P23116		Eukaryotic translation initiation factor 3 subunit A	EIF3A	13669
Q9JIF7		Coatomer subunit beta	COPB	70349
Q3UM45		Protein phosphatase 1 regulatory subunit 7	PP1R7	66385
Q00612		Glucose-6-phosphate 1-dehydrogenase X	G6PD1	14381
P23780		Beta-galactosidase	BGAL	12091
P80314		T-complex protein 1 subunit beta	TCPB	12461
P11983		T-complex protein 1 subunit alpha	TCPA	21454
Q62084		Protein phosphatase 1 regulatory subunit 14B	PP14B	18938
P46935		E3 ubiquitin-protein ligase NEDD4	NEDD4	17999
Q3UHX2		28 kDa heat- and acid-stable phosphoprotein	HAP28	231887
Q9WUM4		Coronin-1C	COR1C	23790
P29416		Beta-hexosaminidase subunit alpha	HEXA	15211
P26516		26S proteasome non-ATPase regulatory subunit 7	PSD7	17463
O08553		Dihydropyrimidinase-related protein 2	DPYL2	12934
P23506		Protein-L-isoaspartate(D-aspartate) O-methyltransferase	PIMT	18537
Q9D1M0		Protein SEC13 homolog	SEC13	110379
Q8BFR4		N-acetylglucosamine-6-sulfatase	GNS	75612
P97825		Hematological and neurological expressed 1 protein	HN1	15374
O35381		Acidic leucine-rich nuclear phosphoprotein 32 family member A	AN32A	11737
Q7TQI3		Ubiquitin thioesterase OTUB1	OTUB1	107260
P42208		Septin-2	SEPT2	18000
O55131		Septin-7	SEPT7	235072
Q8BL97		Serine/arginine-rich splicing factor 7	SRSF7	225027
P48024		Eukaryotic translation initiation factor 1	EIF1	20918
Q9Z1Q5		Chloride intracellular channel protein 1	CLIC1	114584
P54227		Stathmin	STMN1	16765

*extracellular

Supplementary Table S3: Oligonucleotides used for RT-PCR analysis

	Forward	Reverse
Snail1	5′-CTTGTGTCTGCACGACCTGT-3′	5′-CTTCTCACCAGTGTGGGTGC-3′
CD105	5′-GCTACTCATGTCCCTGATCCA-3′	5′-TGGAGTCCCAGAAAGTCAGG-3′
Desmin	5′-TGGAGCGTGACAACCTGATA-3′	5′-AAGGCAGCCAAGTTGTTCTC-3′
GFAP	5′-TAAGCTAGCCCTGGACATCG-3′	5′-GGATCTGGAGGTTGGAGAAA-3′
FN1	5′-AGCAAGCCTGAGCCTGAAGAG-3′	5′-GCGATTTGCAATGGTACAGCT-3′
Vimentin	5′-GGCTGCGAGAGAAATTGC-3′	5′-TCTCTTCATCGTGCAGTTTCTTC-3′
Axin2	5′-CCAAGTGTCTCTACCTCATTTTCCG-3′	5′-GGTTTGTGGGTCCTCTTCATAGC-3′
Akt2	5′-CAAGCGTGGTGAATACATCAAGAC-3′	5′-CTGCTACGGAGAAGTTGTTTAAGGG-3′
Zeb1	5′-TCAGCTGCTCCCTGTGCAGT-3′	5′-AAGGCCTTCCCGCATTCAGT-3′
c-Myc	5′-GCTGGAGATGATGACCGAGT-3′	5′-AACCGCTCCACATACAGTCC-3′
COX2	5′-GGCCATGGAGTGGACTTAA-3′	5′-CTCCACCAATGACCTGATATTT-3′C
15-HPGD	5′-GTTTTACACCCATCAACCATTGC-3′	5′-GGAGGTTAATGGAGCTTTTACGAGT-3′
IL6	5′-CTGATGCTGGTGACAACCAC-3′	5′-CAGAATTGCCATTGCACAAC-3′

All sequences correspond to murine genes

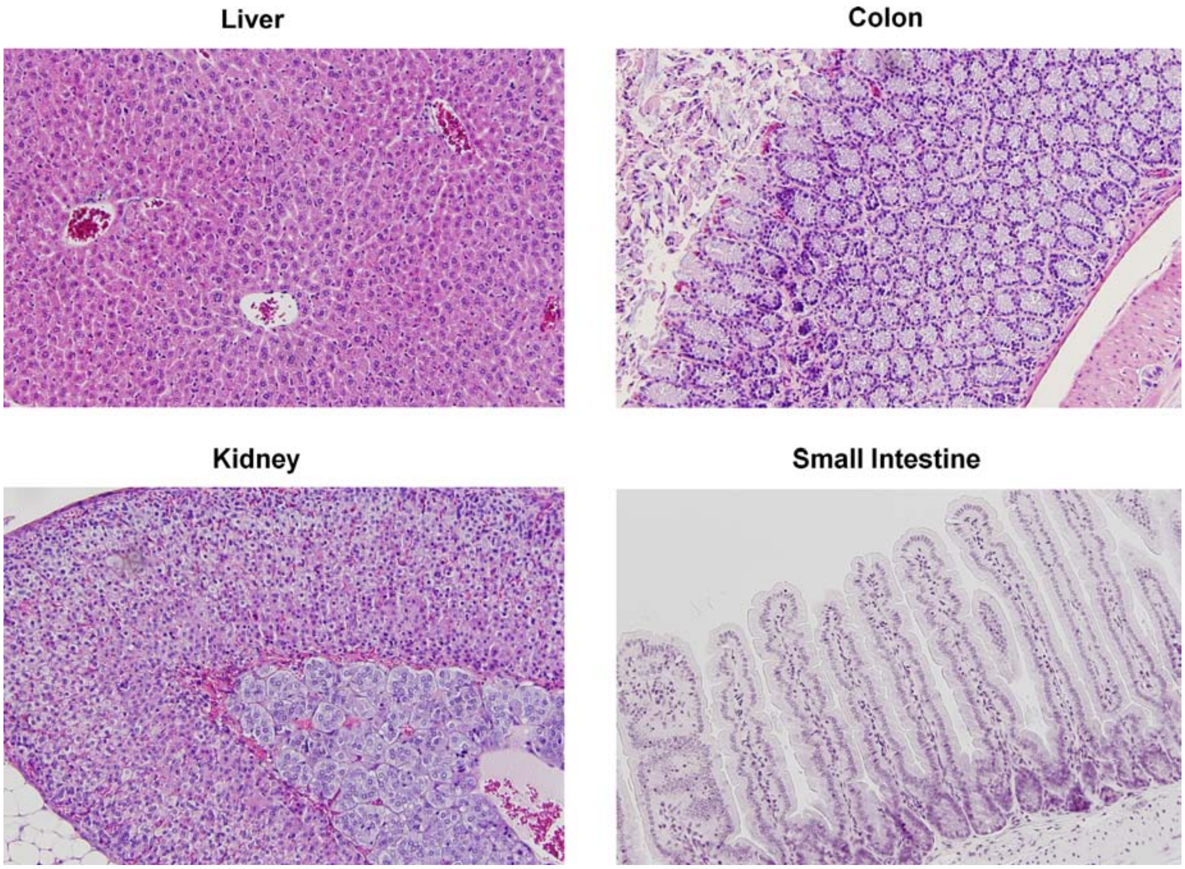

Supplementary Figure S1: Snail1-deficient mice do not present alterations in the liver, colon, kidney or small intestine. The figure shows hematoxylin-eosin staining of sections corresponding to the indicated organs from Snail1$^{Flox/-}$ mice four weeks after tamoxifen injection.

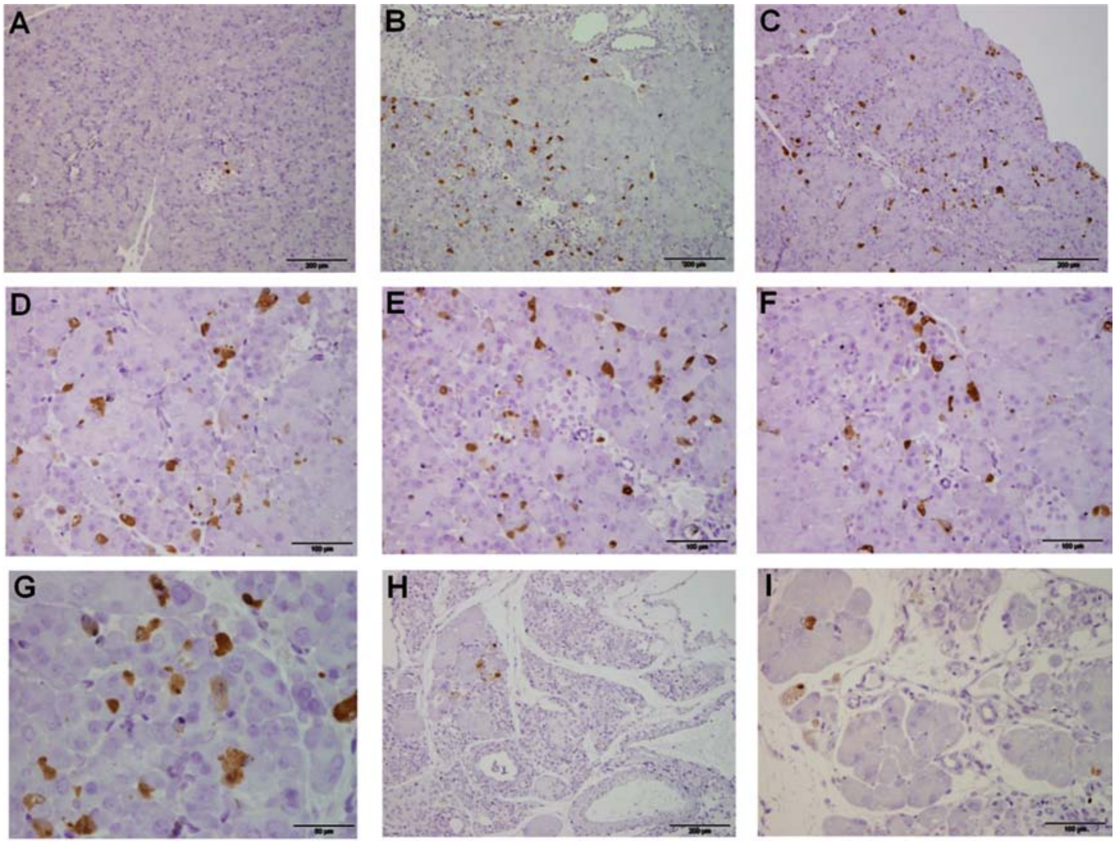

Supplementary Figure S2: Induction of apoptosis in pancreata by Snail1 depletion. Active caspase-3 staining in pancreas from control (Snail1$^{Flox/+}$) (**A**) or Snail1-depleted (Snail1$^{Flox/-}$) (**B**–**I**) mice one week (panels A to G) or two weeks (H–I) after tamoxifen injection.

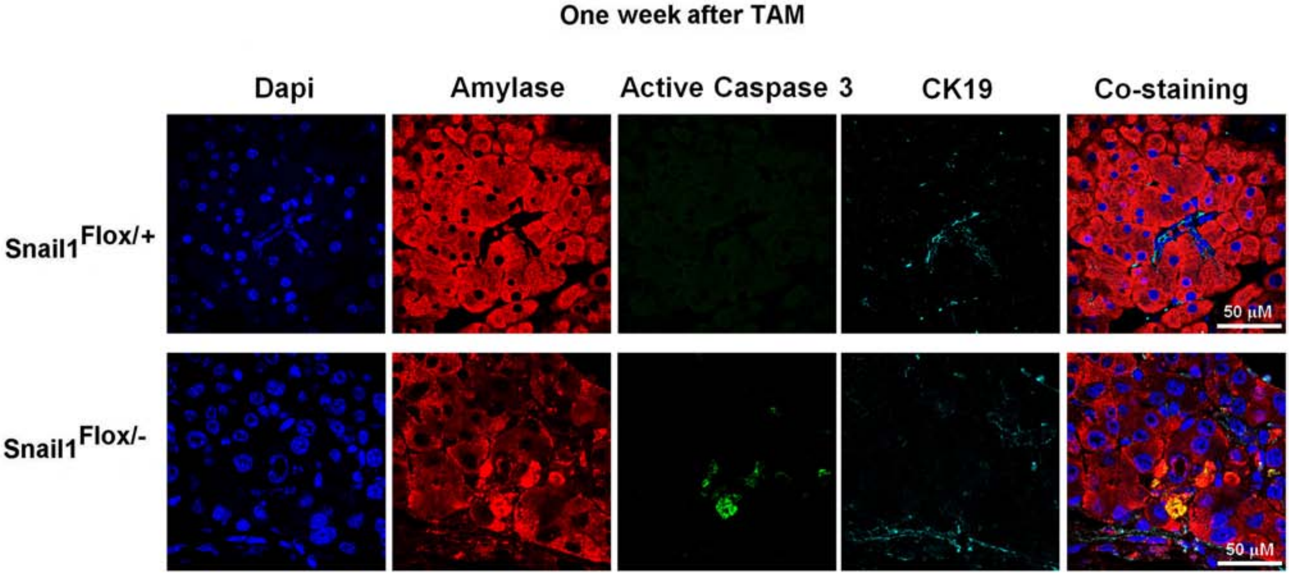

Supplementary Figure S3: Apoptosis in pancreas upon Snail1 depletion takes place in acinar cells. One week after TAM injection control (Snail1$^{Flox/+}$) or Snail1-depleted (Snail1$^{Flox/-}$) pancreas were analyzed for expression of markers of apoptosis (active caspase 3), acinar cells (amylase) or ductal cells (CK19) by immunofluorescence.

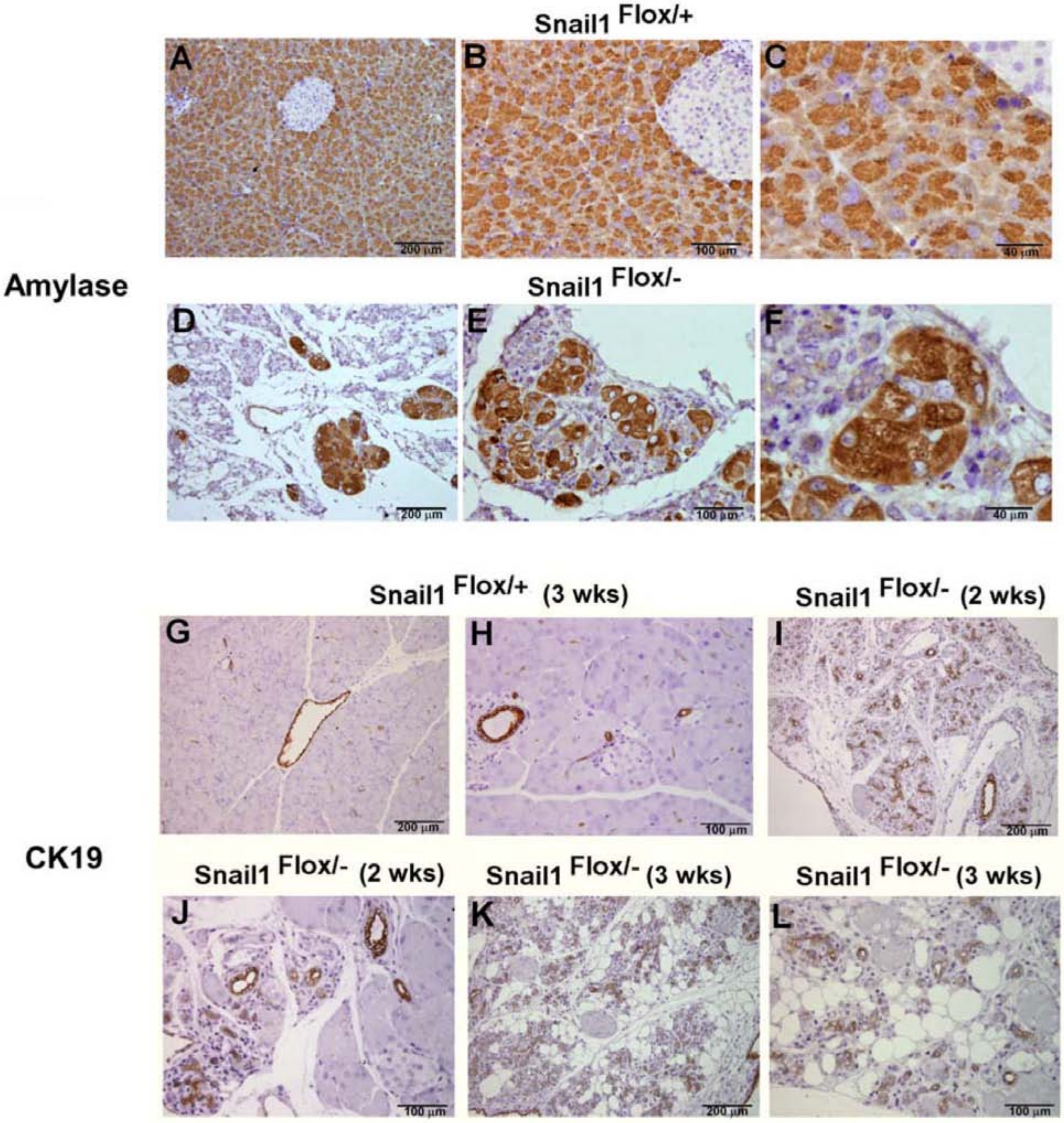

Supplementary Figure S4: Ductal cells are resistant to Snail-depletion. Amylase and CK19 expression was determined in control or Snail1-deficient mice two or three weeks after tamoxifen administration.

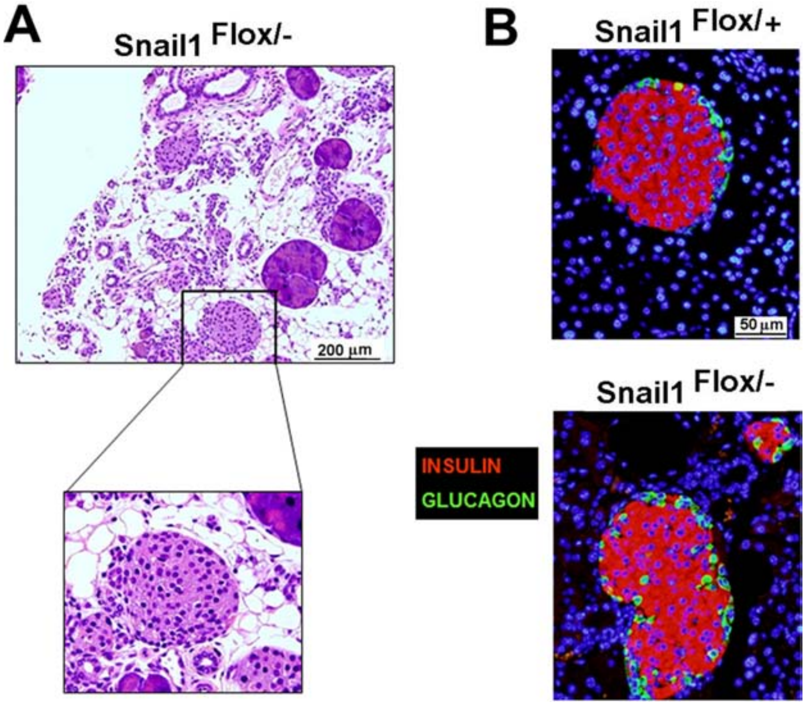

Supplementary Figure S5: Islets are not affected by Snail1 depletion. The figure shows a representative section of a pancreas obtained from a Snail1-deficient mice four weeks after injection of tamoxifen, with the corresponding amplification (**A**), and the immunofluorescence staining of insulin and glucagon in islets obtained from this and from a control (Snail1$^{Flox/+}$) mouse (**B**).

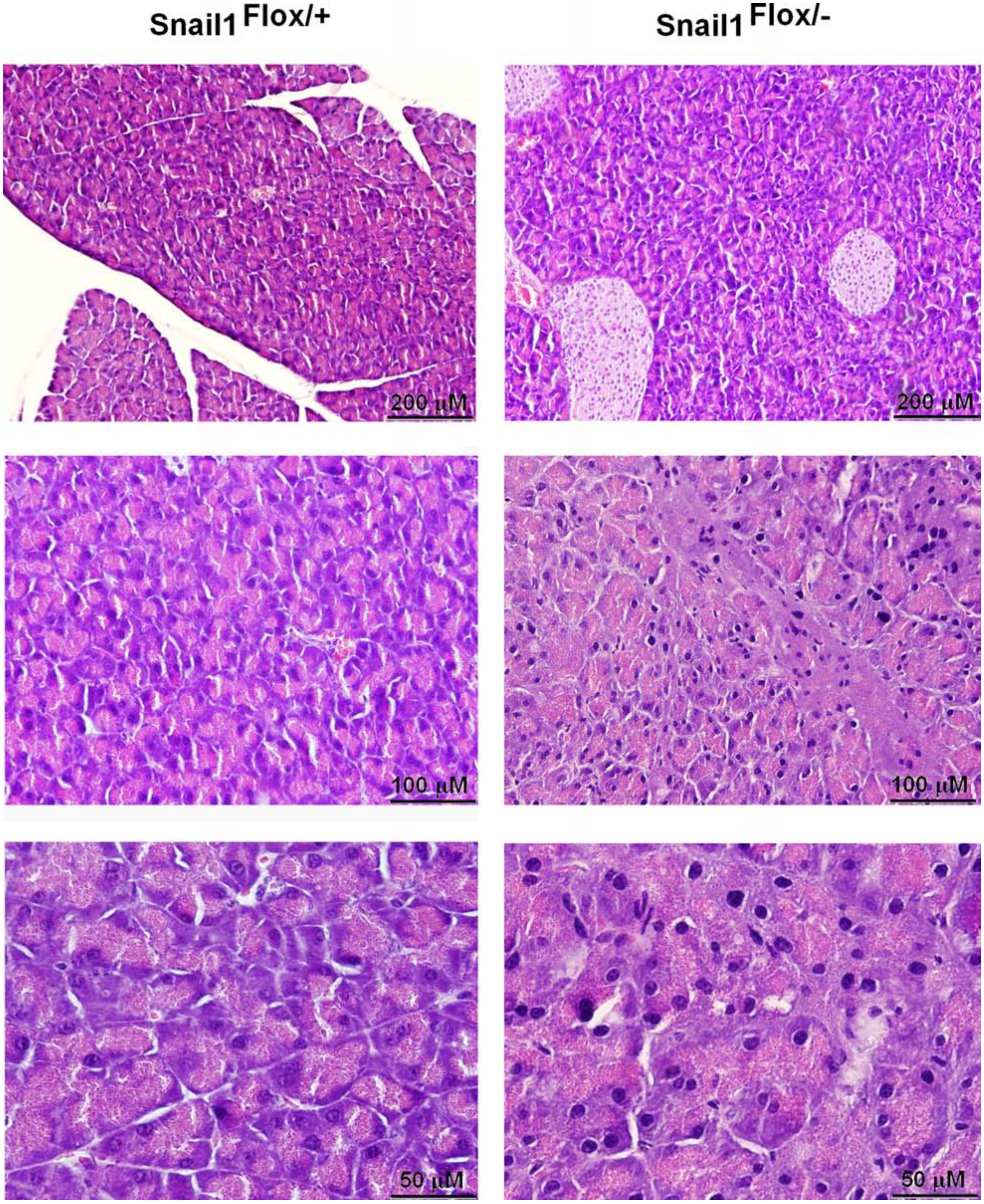

Supplementary Figure S6: Snail1 depletion in acinar cells does not alter pancreas morphology. Two months–old PTF1/p48-Cre-ER, Snail1Flox/− mice were euthanized and pancreas analyzed four weeks after tamoxifen injection. Snail1 Flox/+ mice were used as control. The figure shows a representative micrograph of the organ with magnification.

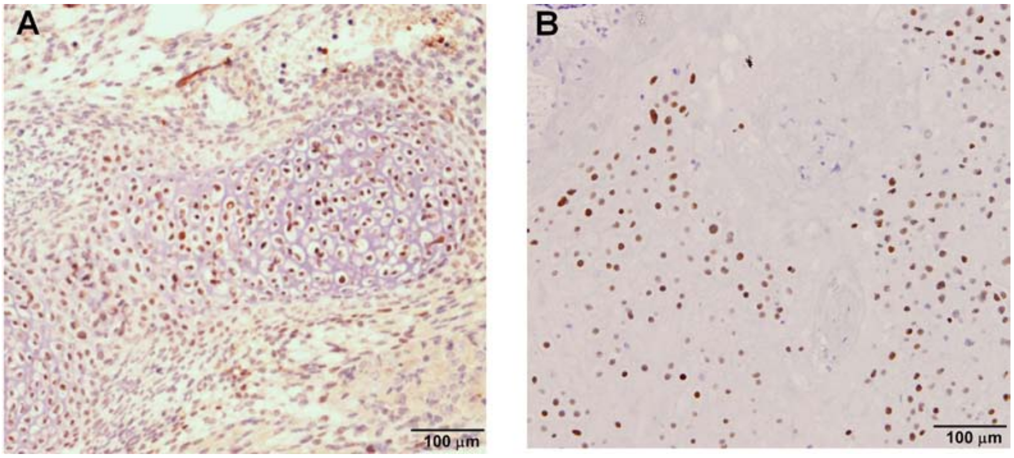

Supplementary Figure S7: Positive controls of Snail1 expression. Expression of Snail1 was determined in paraffin-embedded samples obtained from murine embryos (E15.5) (**A**), or from human placenta (**B**). In embryos expression of Snail1 was high in the cartilage as previously reported (8).

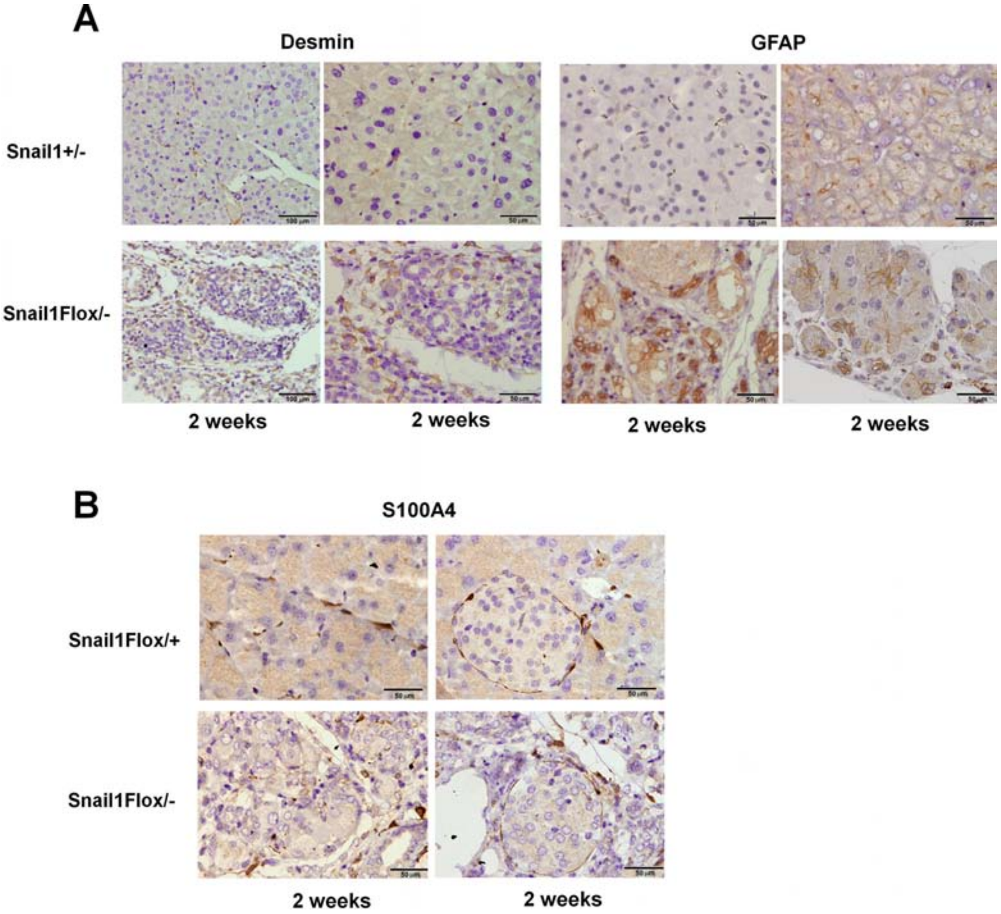

Supplementary Figure S8: Desmin, GFAP or S100A4 stain a different subset of mesenchymal cells than Snail1. Control or Snail1-deficient mice were analyzed for Desmin, GFAP (**A**) or S100A4 (**B**) expression at the indicated times.

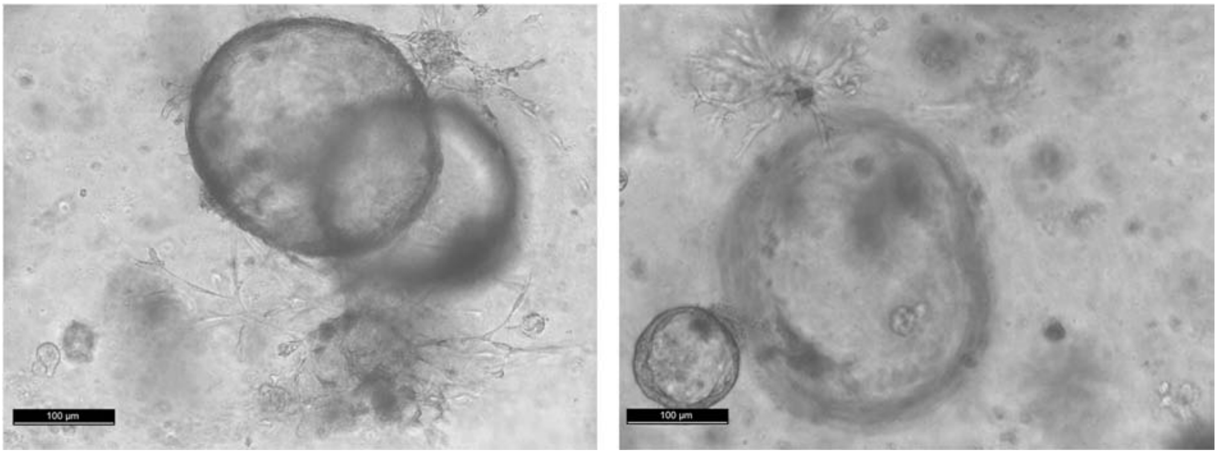

Supplementary Figure S9: Micrograph of a culture of pancreas cells grown in the presence of wild-type PMCs. The morphology of large and medium spheres and co-cultured PMCs is shown.

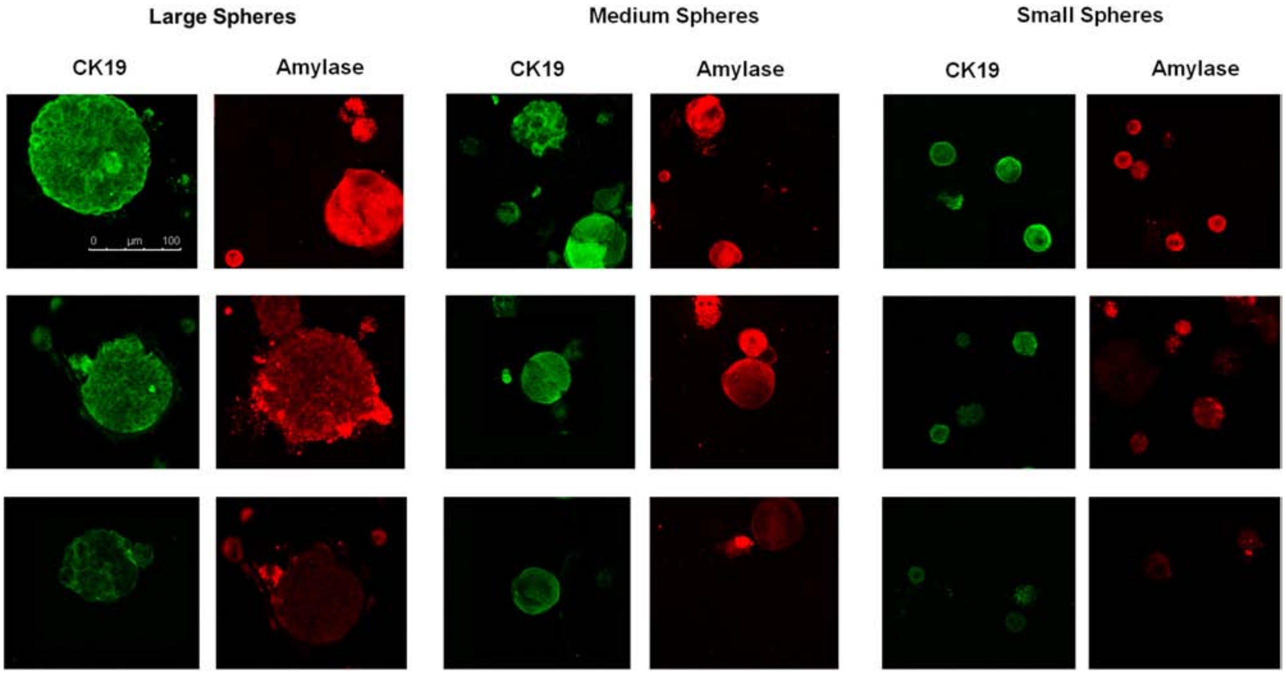

Supplementary Figure S10: Expression of CK19 and Amylase in cultured pancreas spheres. The figure shows large, medium and small spheres stained with Amylase or CK19 antibodies. A representative case of a structure categorized as high, medium or low staining for each makers and each type of structure is shown.

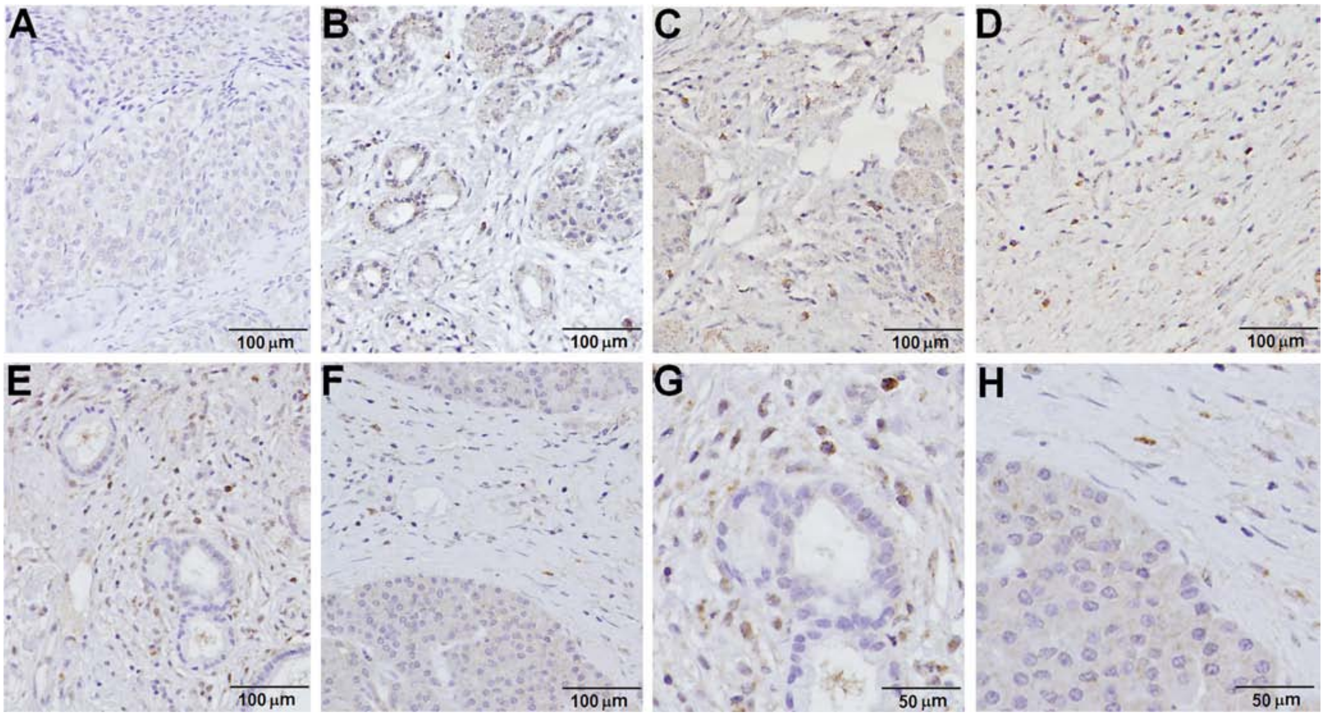

Supplementary Figure S11: Expression of Snail1 in human pancreas tumors. The figure shows the result of Snail1 staining in four preneoplastic lessions (PanIN), two without or with low Snail1 expression (**A** and **B**) and two with Snail1 expression (**C** and **D**), from a total of six analyzed. Snail1 staining in two pancreatic adenocarcinomas is presented in (**E** and **F**). These panels correspond to representative results of the five tumors analyzed, all presenting Snail1 expression in the stroma. Panels (**G** and **H)** are higher magnifications of (E and F), respectively.
